# The role of intracoronary imaging in acute coronary syndromes: OCT in focus

**DOI:** 10.21542/gcsp.2016.36

**Published:** 2016-12-30

**Authors:** Ahmad Samir, Ahmed ElGuindy

**Affiliations:** 1 Aswan Heart Centre, Aswan, Egypt; 2 Faculty of Medicine, Cairo University, Egypt; 3 National Heart and Lung Institute, Imperial College London, London, UK

## Abstract

Optical coherence tomography (OCT) has emerged as a powerful intravascular imaging modality in recent years. The introduction of frequency-domain OCT has simplified the procedure and enabled its safe utilisation in different clinical settings including acute coronary syndromes, where it can determine the mechanism of plaque disruption, thrombus burden, and guide percutaneous coronary intervention. In patients presenting with stent failure (stent thrombosis and instent restenosis), OCT can also be very useful in determining the underlying mechanism and guiding therapy thereafter. This article aims to review the role of OCT in acute coronary syndromes as well as its potential clinical applications.

## Introduction

The vast majority of acute coronary syndromes (ACS) are of atherosclerotic aetiology.^1^ Rupture of a vulnerable atherosclerotic plaque has historically been believed to be the sole mechanism for plaque disruption, which in turn exposes tissue factor to circulating blood triggering coronary thrombosis and ultimately ACS.^2^ However, postmortem studies in the last 25 years revealed that in some patients presenting with myocardial infarction (MI), coronary thrombosis occurred without physical disruption (i.e., rupture or fissuring) of the plaque fibrous cap.^3–5^ The patho-etiology of coronary thrombosis in such scenarios with intact fibrous cap was subsequently studied and two main mechanisms were identified: plaque superficial erosions, and to a much lesser extent, calcific nodules.^3,6–8^

Plaque erosions – currently believed to cause 25–46% of all ACS^4,9^ – were identified pathologically as coronary thrombosis on top of an atherosclerotic plaque with intact fibrous cap, almost always showing endothelial denudation or lacerations at the superficial intimal layer which become primarily composed of smooth muscle cells, and relatively thick fibrous matrix with excess proteoglycans.^3,4,7,8^ The main discriminating microscopic feature between plaque erosion and plaque rupture is the presence of an intact fibrous cap and lack of any communication between the plaque core and the lumen of the vessel (or the luminal thrombus) in the former.^6,8^

**Figure 1.**
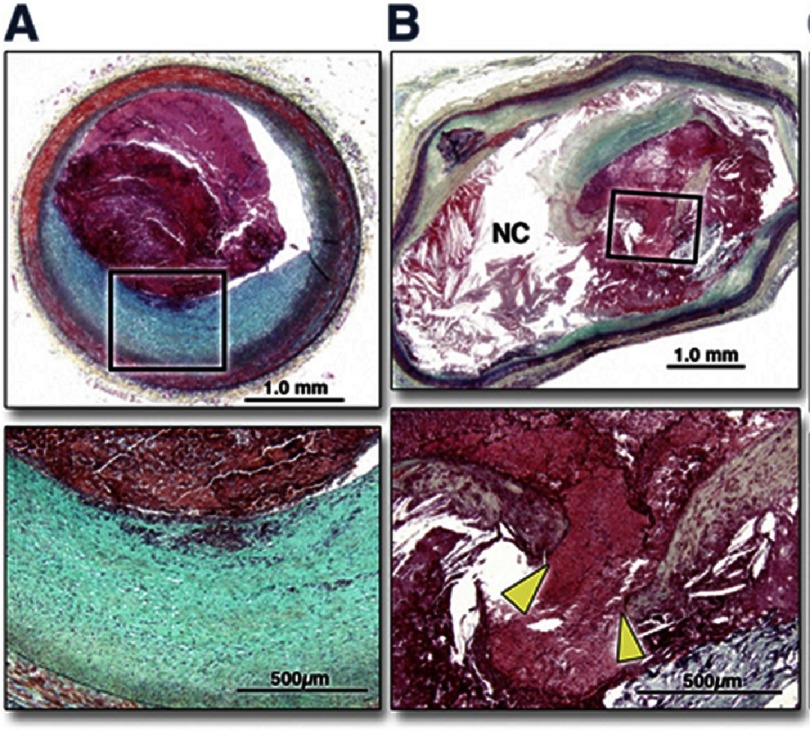
Histomorphological characteristics of plaque erosion and plaque rupture. Cross-sectional images of 2 culprit coronary plaques obtained from the patients with acute coronary syndromes (A,B); the boxed areas in the top panels are magnified in the bottom panels for better histological characterization. The sections are stained with Movat’s pentachrome. (A) The eroded plaque shows subcritical stenosis, an unremarkable necrotic core, and overlying thrombus on an intact fibrous cap. The cap is rich in smooth muscle cells and proteoglycans, and there is minimal inflammation at the base of the thrombus. The plaque does not show any positive remodeling. (B) Conversely, a positively remodeled, critically occlusive atherosclerotic plaque with a cholesterol crystal–rich large necrotic core (NC) covered by a very thin and inflamed fibrous cap, which is disrupted (area between the yellow arrowheads). Smooth muscle cells are visible in the medial layer and thin fibrous cap and minimally present at the base of the neointima. A large thrombogenic necrotic core is in communication with the vessel lumen with an occlusive thrombus.^13^

Calcific nodules, on the other hand, show a highly-calcified matrix causing substantial luminal irregularity with minimal or no lipid core.^3,6,8^ Calcific nodules were encountered in 2–5% of sudden cardiac death (SCD) cases, predominantly affecting the mid right coronary artery (RCA).^8^ However, findings from the PROSPECT study (a large *in-vivo* study utilizing IVUS), suggested that calcific nodules were mostly “innocent bystanders”, posing (by themselves) minimal added risk for ACS for up to 3 years of follow up.^10^ Accordingly, plaque rupture and plaque erosion are frequently considered to be the two main mechanisms for ACS. (Fig. 1)

## *In-vivo* identification of ACS pathogenesis

Limited by its spatial resolution, coronary angiography cannot determine neither the morphology of the culprit lesion, nor the status of the fibrous cap. This means that in every day practice, patients presenting with an ACS frequently end up with their culprit lesion stented regardless of the underlying mechanism for plaque disruption.^11^ Failing to identify the underlying pathology not only meant adopting a “one size fits all” strategy, but also deprives us from understanding the natural history of different types of plaques, and prevents testing targeted therapeutic approaches for different plaque morphologies.^3^ An *in-vivo* vascular imaging modality with sufficient resolution to visualize coronary plaque, demonstrate its composition, identify vulnerable plaques, and define the mode of disruption at time of ACS, would offer the ideal solution.^9,12,13^

Intravascular ultrasound (IVUS), coronary angioscopy (CAS), intravascular optical coherence tomography (IV-OCT), near infrared spectroscopy (NIRS) and intravascular photoacoustic imaging (IVPA) have evolved over the past three decades as tools for intravascular coronary imaging. NIRS currently remains an investigational tool with very limited use in daily clinical practice, while IVPA catheters that can be used safely in-vivo are still under development.^14–20^

Compared to coronary angiography, IVUS and CAS have higher spatial resolution (150–200 µm and 50 µm respectively). However, at this resolution, characterization of plaque morphology and fibrous cap integrity remains challenging.^21^ On the other hand, both NIRS and IVPA offer superb detection of lipid type and plaque composition, with IVPA being capable of identifying active macrophages and determining matrix metalloproteinase activity. However, NIRS lacks depth resolution and is unable to define the exact location or amount of the lipid pool, and IVPA has very limited ability to define the anatomical details of the arterial wall. This has led to the current perception of both modalities as hybrid technologies that require superimposition on IVUS images.^19,20^

## The introduction of OCT

Introduction of OCT as a novel technique for intravascular imaging is frequently considered the real beginning for accurate *in-vivo* characterization of coronary plaques and the mechanism of their disruption.

**Figure 2.**
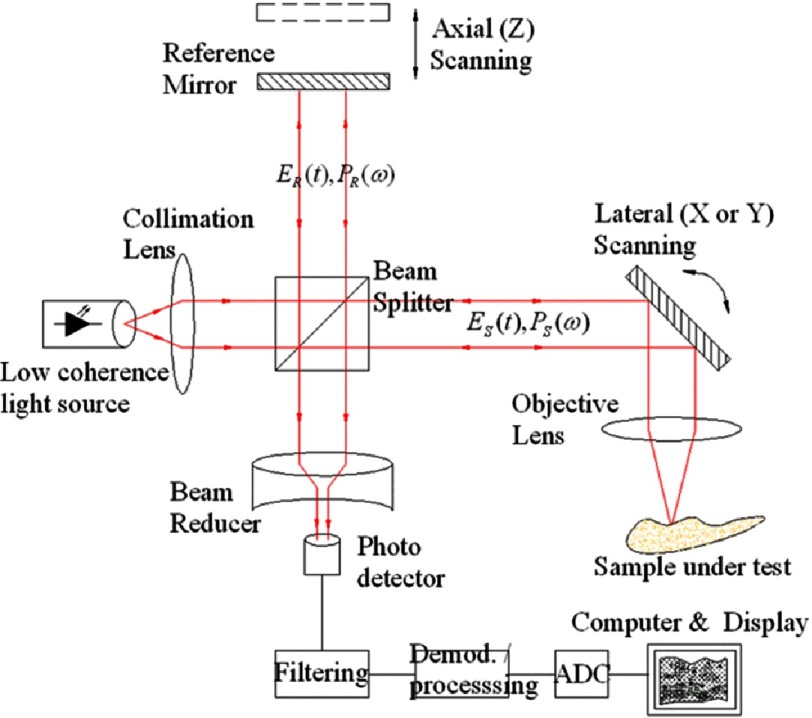
Physical principle of operating OCT imaging.

Optical coherence tomography is a novel imaging technique that can deliver three-dimensional images from an optical scattering medium (like the arterial wall). It employs near infra-red light rays and depends on interferometric principles.^22–24^ Light in OCT systems comes from ultra-short pulsed lasers capable of emitting a wide range of wavelengths over ∼100 nm. The emitted light beam is then broken into two arms: one goes to the item of interest, and a reference arm going to a semi-reflective mirror. During processing, combination of reflected light from both beams gives an interference pattern if light has travelled similar optical distance. Reflectivity profile of the item of interest can be made by scanning the mirror of the reference light beam, where structures reflecting more light create more interference and so forth, while light outside the short coherence length will not cause interference. The obtained reflectivity profile (the A-scan) gives details about location and spatial dimensions of the structure(s) of interest. Subsequently, a series of those acquired A-scans are laterally combined to obtain a cross-sectional tomograph (the B-scan).^24–27^ This is the main principle behind the time-domain (TD) OCT (Fig. 2: Physical principle of operating OCT imaging). The more recent frequency-domain (FD) OCT enhanced both the applicability and resolution, significantly reduced motion artifacts, and improved signal-to-noise ratio. The most important advancement from TD to FD-OCT was abolishing the need for proximal occlusion, which for many years hindered the use of OCT imaging in day-to-day clinical practice.^28–30^

**Figure 3.**
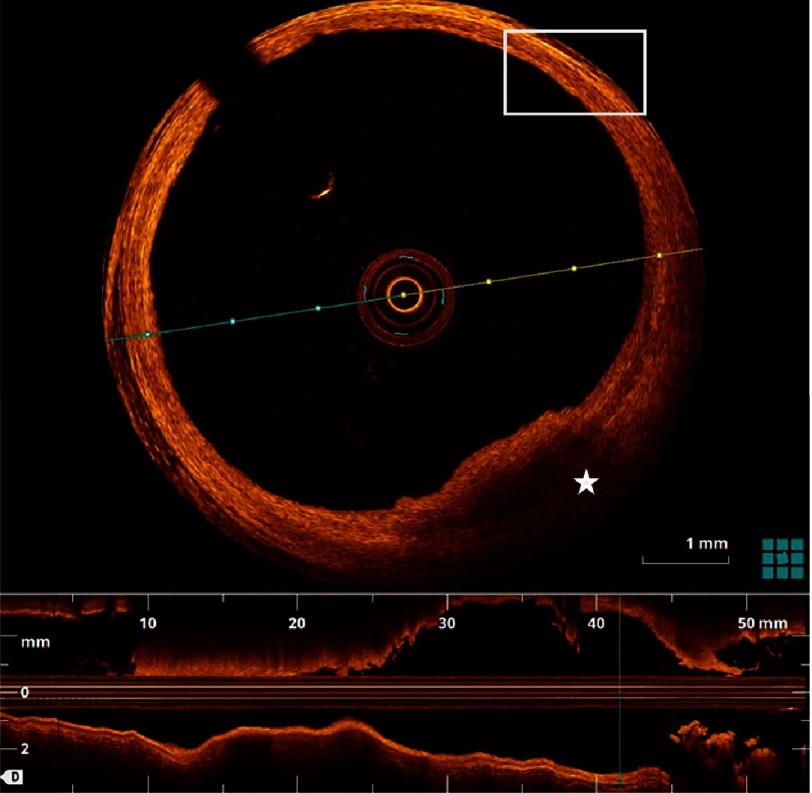
Lipid atherosclerotic plaque. Lipid plaques appear as poor signal regions with ill-defined borders (star), in contrast to the healthy region (box) composed of signal rich, signal poor and signal rich layers representing intima, media and adventitia respectively.

**Figure 4.**
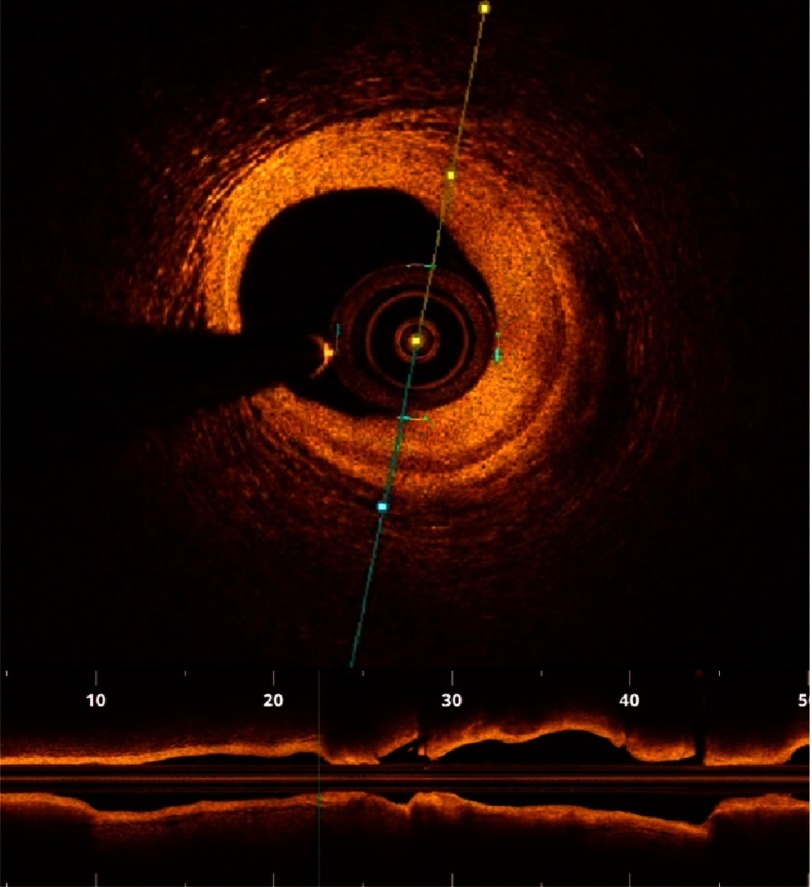
Fibrous atherosclerotic plaque. Fibrous plaques appear as homogenous high signal regions (pathological intimal thickening) displacing media externally.

**Figure 5.**
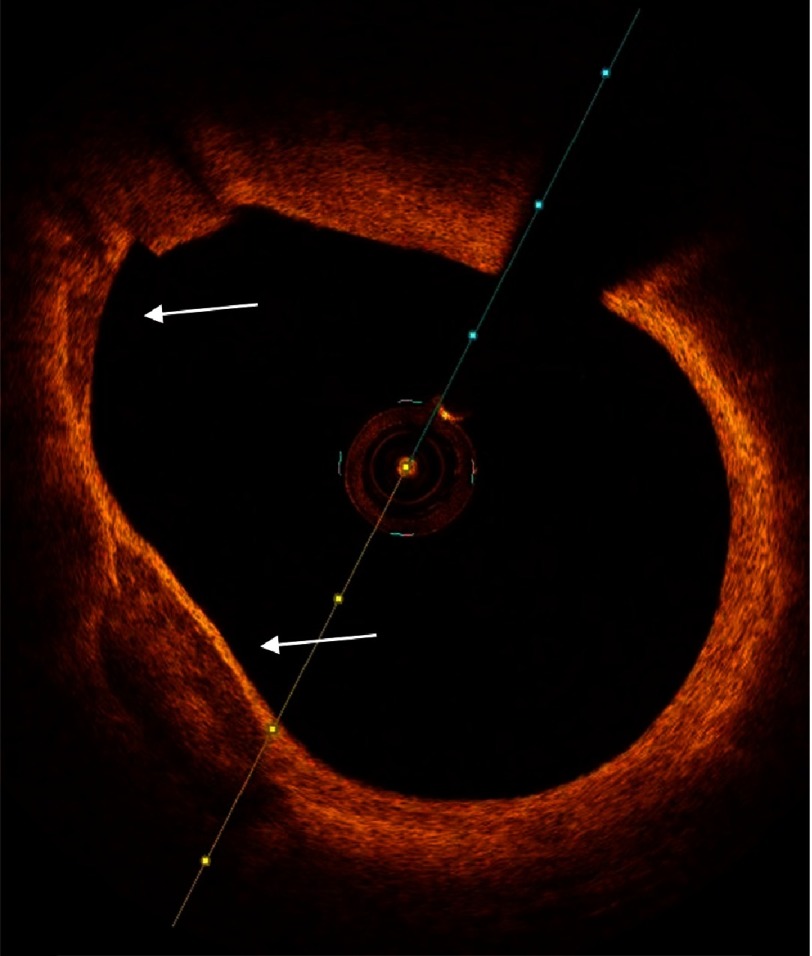
Calcific atherosclerotic plaques. Calcific plaques appear as signal poor regions with sharply defined borders (arrows).

**Figure 6.**
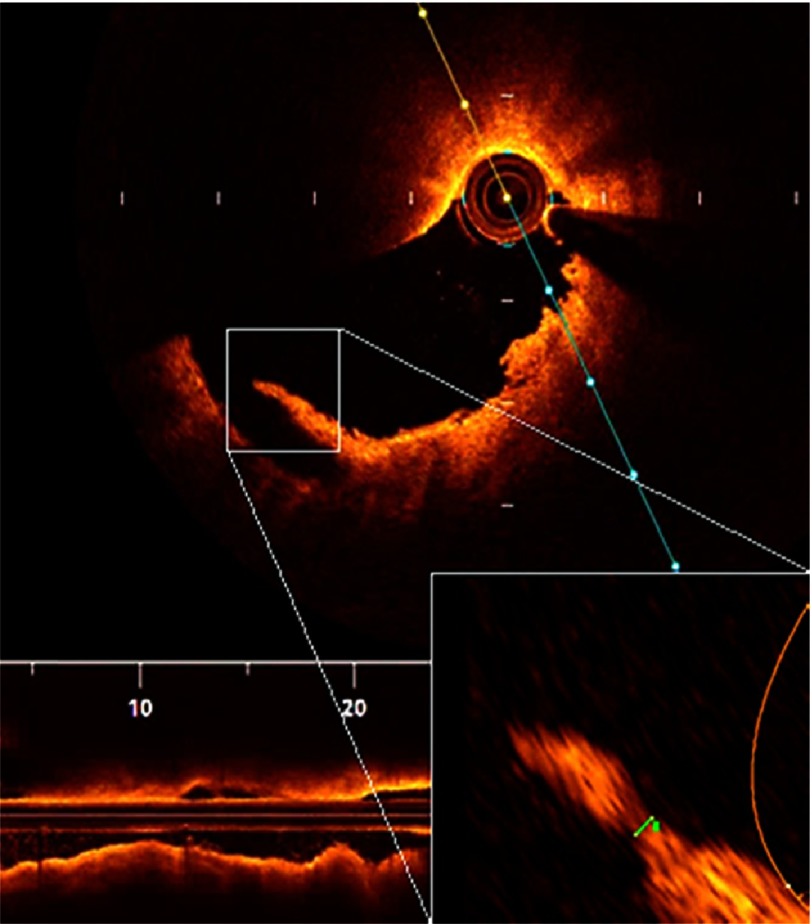
Measuring fibrous cap thickness. After magnifying the small box, the remnant of the ruptured fibrous cap was measured and was 52 micrometers.

**Figure 7.**
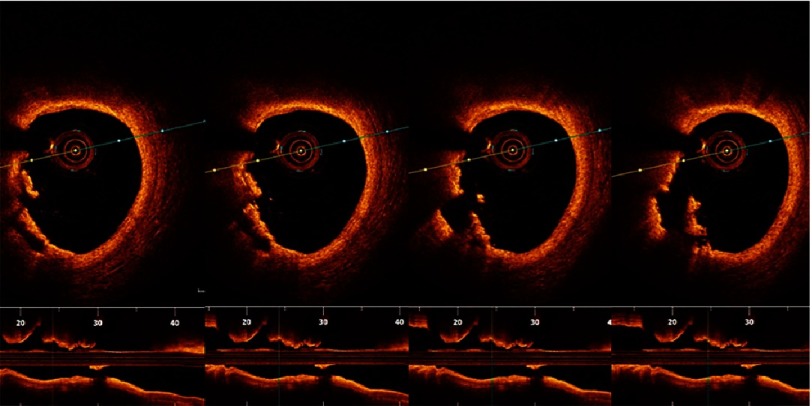
Plaque rupture. Serial cuts in a pullback in LAD showing discontinuity of the plaque cap with subsequent formation of a cavity.

**Figure 8.**
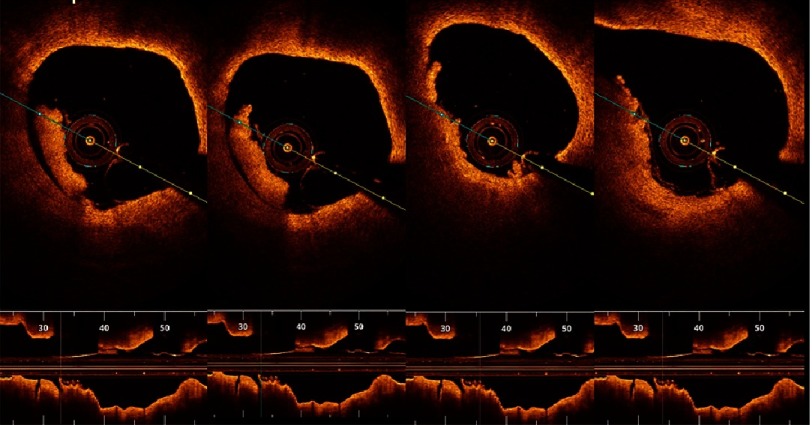
Plaque erosion. Serial cuts in a pullback in proximal RCA showing thrombus formed at site of plaque erosion with eroded intima yet intact fibrous cap.

**Figure 9.**
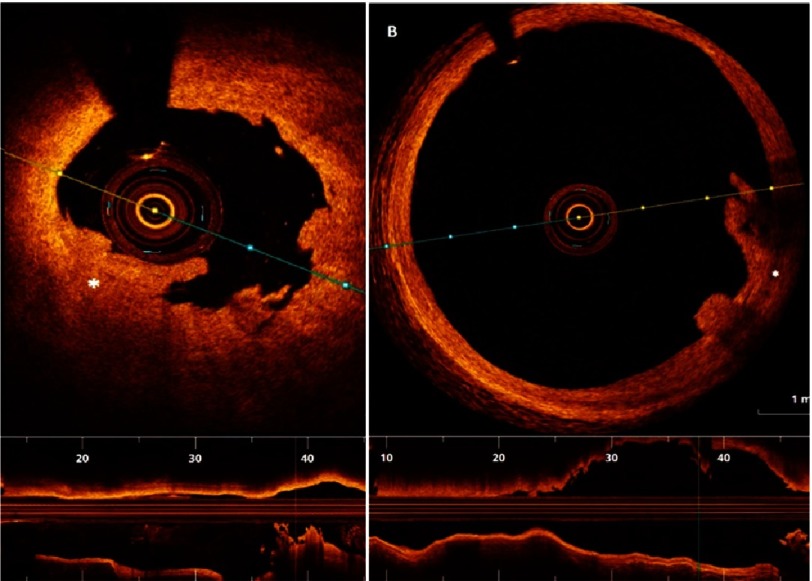
White thrombus (in “A”), and red thrombus (in “B”). White thrombus is typically characterized by high signal low backscattering (A), while red thrombus (B), is characterized by lower signal with high backscattering.

OCT offers a micron-scale resolution (10 µm) which enables accurate characterization of the plaque content with the ability to differentiate between fibrous, lipid and calcific plaques (Figs. 3–5). OCT also allows clear visualization of the plaque fibrous cap as well as precise measurement of its thickness, which is a critical marker of impending rupture and plaque vulnerability (Figs. 6–8). To date, precise *in-vivo* measurement of the thickness of fibrous cap is not possible by any other intracoronary imaging modality.^31^ Compared to IVUS, OCT is significantly superior in its ability to detect luminal and mural thrombi, discriminate between RBC-rich red thrombi (high backscattering and high beam attenuation) and platelet-rich white thrombi (low backscattering and low attenuation) (Fig. 9). One of the main advantages of OCT’s unprecedented resolution is its remarkable accuracy in detecting plaque cap rupture; thus differentiating between disrupted plaques with ruptured and those with intact fibrous caps.^21,32^

In a study comparing OCT, IVUS and CAS in the assessment of culprit lesions in patients with acute myocardial infarction cases, Kubo et al. found that OCT had significantly higher accuracy for detecting plaque rupture (73% vs. 40% vs. 43% respectively, *p* = 0.021), plaque erosion (23% vs. 0% vs. 3% respectively, *p* = 0.003) and coronary thrombi (100% vs. 33% vs. 100% respectively, *p* < 0.001)^31^ (Table 1).

**Table 1:** Comparison of characteristics of novel intra coronary imaging modalities.

	Gray-scale IVUS	CAS	OCT
Imaging source	Ultra-sound	Visible light	Near infra-red light
Resolution (µm)	150–200	50	10–20
Penetration depth (mm)	5	none	2
Lipid pool	^*^	^**^	^***^
Calcium	^***^	none	^***^
Fibrous cap	^*^	^**^	^***^
Macrophages	none	none	^*^
Microvascularizations	none	none	^**^
Thrombus	^*^	^***^	^***^

**Notes.**

* modest

** good

*** excellent identification

### OCT characterization of atherosclerotic plaques

**Figure 10.**
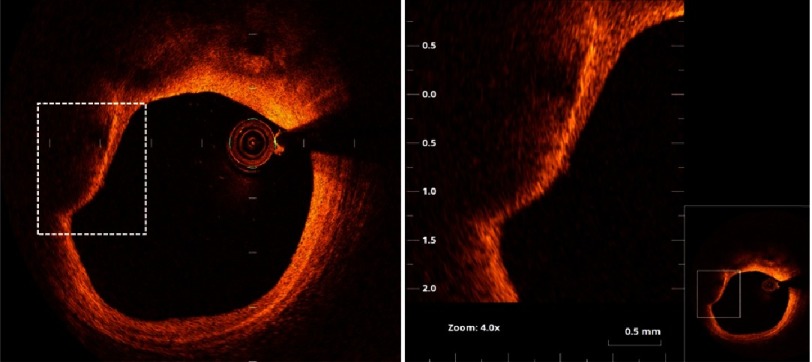
Thin cap fibro-atheroma. The right panel is magnification of the square in the left panel (as shown in thumbnail). A plaque with large ill-defined lipid pool and thin fibrous cap (measuring 58 µm). The arrow points to accumulation of macrophages (the bright line) beneath the thin cap.

Owing to its superb image quality, cumulative knowledge about the OCT features of ruptured plaques and eroded plaques had accumulated over the past few years. Ruptured plaques are typically characterized by a large lipid core that appears as signal-poor regions with poorly defined borders and rapid signal drop off. The fibrous cap separating the plaque core from the arterial lumen is typically thin. The term “thin cap fibro-atheroma (TCFA)” is used to describe plaques with a fibrous cap that is <65 µm thick and a lipid pool extending through 2 or more quadrants, and is considered the primary substrate for plaque rupture (Fig. 10). Macrophages and neovascularization are typically found more frequently in ruptured plaques or TCFAs and rarely (if any) encountered in eroded plaques. Macrophage clusters appear in OCT as a shimmering line or signal-rich distinct glistening region compared to its surrounding structure and cause strong signal attenuation.^25^ Activated macrophages are believed to correlate with the degree of inflammation in the plaque cap especially with the release of matrix metallo-proteinases (MMP) that accelerate cap degradation and thus rupture.^33–35^ In a study to validate OCT detection of macrophages against pathology, OCT was found to be 100% sensitive and specific in identifying macrophages in fibrous caps that had >10% CD68 staining.^36^

**Figure 11.**
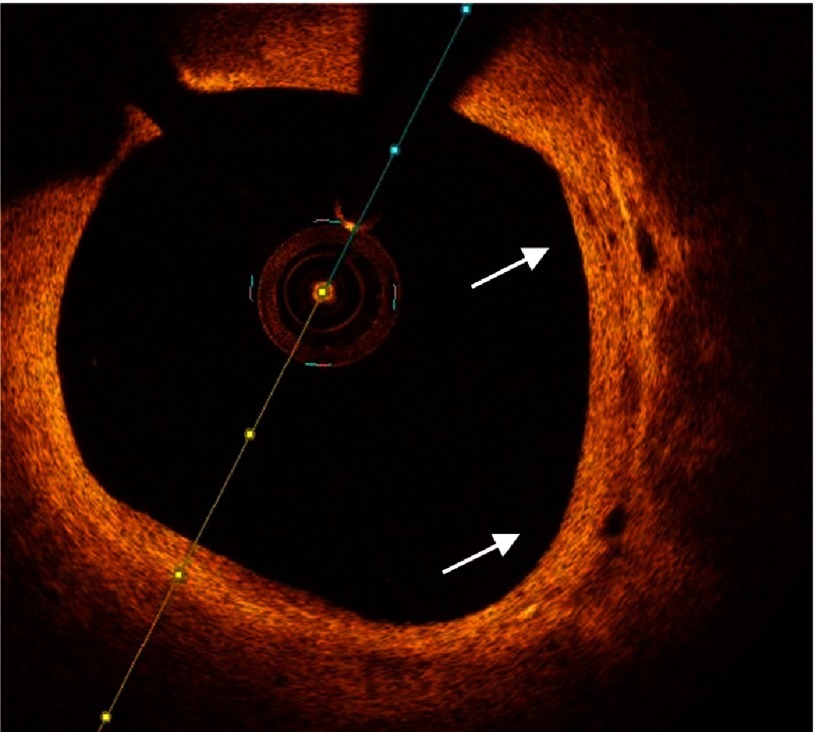
Neovascularization of atherosclerotic plaque (arrows).

Neovascularization appears as sharply delineated signal-free voids located on the intimal side of large plaques, and is usually present in at least 3 successive frames.^24,25,37^ Neovascularization is a substrate for intra-plaque hemorrhage (which can cause sudden increase in plaque size), and is strongly associated with local inflammation and plaque vulnerability. (Fig. 11).^3,38,39^

On the other hand, eroded plaques usually are predominantly fibrous, with minimal lipid content. Compared to ruptured plaques, eroded plaques are typically characterized by relatively thicker fibrous caps. The higher OCT light signal of such fibrotic plaques is usually correlated with the high proteoglycan content. Macrophages and neovascularization are only sparsely detected in eroded plaques. It is important to recognize that the current resolution of OCT is still insufficient to visualize endothelial cells and detect such denudation. For this reason, the term “*disrupted plaque with intact fibrous plaque (IFC)*” rather than plaque erosion is preferable.^40^

*In-vivo* identification of erosion-prone plaques before they get disrupted and cause ACS remains a great challenge. To the best of our knowledge, there are currently no markers that can predict erosion of a stable fibrotic, thick-capped plaque. This means that at least one third of catastrophic acute coronary events are impossible to predict despite the major advances in coronary imaging, which remains an important area for future research.

### Translating knowledge into practice

Data from small observational studies suggest that culprit plaque morphology could have a strong impact on the clinical outcomes of AMI patients.^12^ In a study involving 72 consecutive anterior AMI patients, MIs induced by plaque rupture were found to cause more myocardial damage and poorer functional recovery compared to those caused by plaque erosions, even after successful primary angioplasty.^41^ In another study, culprit plaques with ruptured fibrous caps (RFC) were associated with larger infarct size than those with intact fibrous cap (IFC).^42^

Despite these differences, acute myocardial infarctions are currently treated in a standardized fashion regardless of the underlying mechanism, simply because they all look alike in coronary angiography. The primary treatment objective in patients with STEMI is rapid restoration of TIMI III flow in the culprit vessel; usually (and preferably) achieved with stenting. It is currently unclear whether information about the plaque phenotype can/should alter this strategy. Patients with intact fibrous caps and non-obstructive lesions after thrombus aspiration (or thrombolysis), in particular, represent a distinct group of patients in whom a no-stent strategy may be appealing. This hypothesis was tested in a small study by Prati et al. where – at the discretion of the surgeon – 12 out of 31 patients presenting with acute STEMI and intact fibrous caps with non-obstructive underlying plaques were treated by lone thrombus aspiration, thus sparing patients the added – albeit small – risk of a stent.^13^ The other 19 patients were treated in the conventional manner (i.e., received a second-generation drug-eluting stent). After a follow up period of >2 years, no patients in the lone thrombectomy group and only one patient in the conventional therapy group required repeat revascularization. These interesting results warrant further research into the possible advantages and disadvantages of adopting a treatment strategy that is tailored to the plaque phenotype and severity of residual luminal stenosis.^9,13^

From a technical perspective, OCT can be very helpful in planning and optimizing PCI results. Factors such as stent diameter and length, landing zones, lesion characteristics requiring special preparation (such as severe calcification), stent under-expansion, malapposition, and edge dissection are amongst the parameters that can be readily identified by OCT. In ILUMIEN I study which enrolled 418 patients, pre-stenting OCT imaging modified PCI strategy in 55% of cases. After PCI was completed, further intervention was deemed necessary by the operator after OCT imaging in 25% of cases (malapposition in 14.5%, stent under-expansion in 7.6%, and stent edge dissection in 2.7%).^43^

In another multicenter study randomizing 240 NSTE-ACS patients to OCT versus angiography-guided intervention, OCT changed procedural strategy in 50% of cases in the OCT group. Post procedural FFR values were significantly higher in the OCT-guided group (0.94 ± 0.04 versus 0.92 ± 0.05, *p* = 0.005). Post-procedure OCT imaging revealed under-expansion in 42%, stent malapposition in 32%, stent edge dissection in 37.5% of cases. OCT use was associated with the use of more contrast, and longer procedural times, but the rates of acute kidney injury and overall procedural complications were identical both groups.^44^ Whether such optimization translates into better outcomes remains to be tested in larger trials with hard clinical endpoints.

### Understanding, preventing and treating stent failure

**Figure 12.**
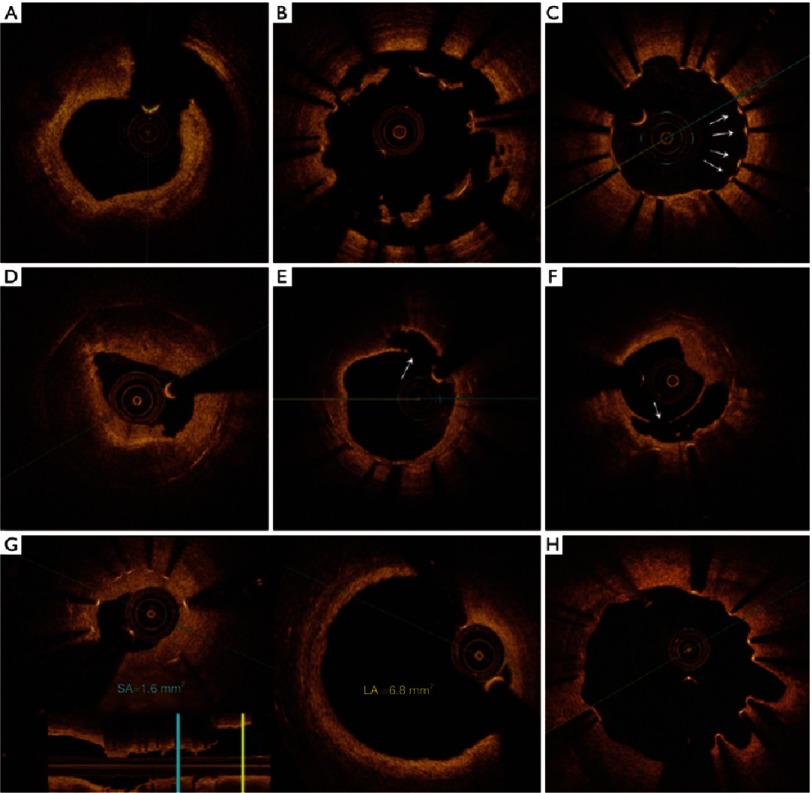
OCT depicting different mechanisms of stent thrombosis. Acute stent thrombosis with underlying stent edge dissection (A); Subacute stent thrombosis with underlying major malopposition (B); Late stent thrombosis and underlying isolated uncovered struts(C); neoatherosclerotic plaque in a case of very late stent thrombosis (D); Rupture of a neoatherosclerotic lesion (E and F); underexpanded stent with measurements of stent area and reference luminal area (H). Souteyrand et al.^55^

With the current generation of drug eluting stents, the incidence of stent thrombosis is becoming very low (<1%).^45–47^ However, these few events are often catastrophic with reported mortality rates as high as 40%.^48,49^ Important procedural risk factors for stent thrombosis include stent under-expansion and malapposition, edge dissection, and residual disease proximal or distal to the stent.^47,50^ Other mechanisms implicated in stent thrombosis include neoatherosclerosis (with or without rupture), isolated uncovered struts, and evaginations related to late positive remodeling (Fig. 12).^51–53^

The treatment of patients presenting with stent thrombosis can be substantially different depending on the underlying mechanism; e.g., balloon dilatation with gross underexpansion, stent-in-stent for neoatherosclerosis, or thrombus aspiration followed by prolonged dual antiplatelet therapy (frequently with a 24-48 hour infusion of IV GP IIb/IIIa inhibitors in the acute phase) with isolated uncovered struts.^47,48,54^ OCT is able to identify the underlying mechanism in the vast majority of instances, and is therefore a perfect diagnostic tool to guide therapy in such critical situations.^55^

The incidence of instent restenosis has declined substantially with the introduction of drug eluting stents. The rates of angiographic and clinically relevant restenosis with the newer generation drug eluting stents are 12% and 5% respectively.^54^ Contrary to bare metal stents where the main mechanism for restenosis is neointimal hyperplasia, the predominant mechanism for restenosis with drug eluting stents is accelerated neoatherosclerosis. Stent under-expansion - defined as minimum stent area <70% of reference luminal area - has been shown to be a strong predictor of stent failure (instent restenosis or stent thrombosis).^56–59^

The European Society of cardiology guidelines for revascularization has therefore recommended the use of IVUS or OCT in the assessment and management of stent failure as class IIa.^60^ Because of its 10x-to-15x higher resolution compared to IVUS, OCT is progressively becoming the modality of choice for this indication.^46,55,57^

## Conclusion

OCT has emerged as an exciting and powerful intravascular imaging modality. With the introduction of FD-OCT, image acquisition has become a simple and safe task that can be accomplished within a few minutes. Because of its high resolution, OCT is able to provide immediate in-vivo information about the mechanism of plaque disruption, mode of stent failure, and can guide interventions - particularly in complex anatomies. Incorporating the multitude of information provided by OCT in day-to-day decision making, understanding its impact on clinical outcomes, and determining its cost effectiveness in different scenarios remain to be tested in larger-scale clinical studies.
